# Patient experience with chronic obstructive pulmonary disease: a nationally representative demonstration study on quality and cost of healthcare services

**DOI:** 10.3389/fpubh.2023.1112072

**Published:** 2023-06-15

**Authors:** Seyyed-Hadi Ghamari, Farnam Mohebi, Mohsen Abbasi-Kangevari, Soheil Peiman, Besharat Rahimi, Naser Ahmadi, Yousef Farzi, Shahedeh Seyfi, Nazila Shahbal, Mitra Modirian, Mehrdad Azmin, Hossein Zokaei, Maryam Khezrian, Roya Sherafat, Mohammad-Reza Malekpour, Shahin Roshani, Negar Rezaei, Mohammad Javad Fallahi, Maryam Haddadzadeh Shoushtari, Zahra Akbaripour, Shahab Khatibzadeh, Saeid Shahraz

**Affiliations:** ^1^Non-Communicable Diseases Research Center, Endocrinology and Metabolism Population Sciences Institute, Tehran University of Medical Sciences, Tehran, Iran; ^2^Haas School of Business, University of California, Berkeley, CA, United States; ^3^Department of Internal Medicine, AdventHealth Orlando Hospital, Orlando, FL, United States; ^4^Heller School of Social Policy and Management, Brandeis University, Waltham, MA, United States; ^5^The Netherlands Cancer Institute (NKI), Amsterdam, Netherlands; ^6^Endocrinology and Metabolism Population Sciences Institute, Tehran University of Medical Sciences, Tehran, Iran; ^7^Thoracic and Vascular Surgery Research Center, Shiraz University of Medical Sciences, Shiraz, Iran; ^8^Department of Internal Medicine, Shiraz University of Medical Sciences, Shiraz, Iran; ^9^Air Pollution and Respiratory Diseases Research Center, Ahvaz Jundishapur University of Medical Sciences, Ahvaz, Iran; ^10^Razi University Hospital, Guilan University of Medical Sciences, Guilan, Iran; ^11^Tufts Medical Center, Institute for Clinical Research and Health Policy Studies, Boston, MA, United States

**Keywords:** COPD, continuity of patient care, healthcare utilization, patient journey, quality of care, standard of care

## Abstract

**Introduction:**

Due to insufficient data on patient experience with healthcare system among patients with chronic obstructive pulmonary disease (COPD), particularly in developing countries, this study attempted to investigate the journey of patients with COPD in the healthcare system using nationally representative data in Iran.

**Methods:**

This nationally representative demonstration study was conducted from 2016 to 2018 using a novel machine-learning based sampling method based on different districts’ healthcare structures and outcome data. Pulmonologists confirmed eligible participants and nurses recruited and followed them up for 3  months/in 4 visits. Utilization of various healthcare services, direct and indirect costs (including non-health, absenteeism, loss of productivity, and time waste), and quality of healthcare services (using quality indicators) were assessed.

**Results:**

This study constituted of a final sample of 235 patients with COPD, among whom 154 (65.5%) were male. Pharmacy and outpatient services were mostly utilized healthcare services, however, participants utilized outpatient services less than four times a year. The annual average direct cost of a patient with COPD was 1,605.5 USDs. Some 855, 359, 2,680, and 933 USDs were imposed annually on patients with COPD due to non-medical costs, absenteeism, loss of productivity, and time waste, respectively. Based on the quality indicators assessed during the study, the focus of healthcare providers has been the management of the acute phases of COPD as the blood oxygen levels of more than 80% of participants were documented by pulse oximetry devices. However, chronic phase management was mainly missed as less than a third of participants were referred to smoking and tobacco quit centers and got vaccinated. In addition, less than 10% of participants were considered for rehabilitation services, and only 2% completed four-session rehabilitation services.

**Conclusion:**

COPD services have focused on inpatient care, where patients experience exacerbation of the condition. Upon discharge, patients do not receive appropriate follow-up services targeting on preventive care for optimal controlling of pulmonary function and preventing exacerbation.

## Introduction

Chronic obstructive pulmonary disease (COPD) is a progressive, debilitating lung disease contributing to 926.1 disability-adjusted life-years (DALYs) per 100,000 population in 2019, worldwide (1). The World Health Organization deduced that unless proper measures are taken, COPD will turn the disease to the third most leading cause of death by 2030 (2). On the other hand, COPD is a chronic disease with catastrophic aftermaths, which imposes high hospitalizations, healthcare utilization, and direct and indirect costs on patients and healthcare systems (3–5). The total economic cost of COPD according to American Lung Association report in 2019 is close to $50 billion each year, including $29.5 billion for direct health care expenditures, $12.4 billion for indirect mortality costs, and $8.0 billion for indirect morbidity costs (6).

Commensurate with the global scale, COPD has yet remained as a significant public health concern in Iran, as roughly 380,000 DALYs were attributed to COPD from 1990 to 2019 in Iran (7). In addition, it has been estimated that the annual hospitalization cost per patient with COPD was 865 US dollars in Iran (8). Therefore, assessing the trends in healthcare quality and costs for COPD as a globally high-volume and high-cost disease would shed light on the current disease status. This would potentially empower policymakers to seize any opportunities to lower the costs and increase the quality of care (9).

Investigating utilization, quality, and healthcare costs at national level often requires relatively high budgets and expenditures, limiting investigators’ capabilities, especially regarding sampling methods. Addressing the limitations of previous sampling methods, we established a cohort study with a novel machine-learning based sampling method for evaluating the medical care. Iran Quality of Care Medicine Program (IQCAMP) is series of longitudinal surveys focusing on the patient-experience with healthcare services, which measure multiple patient-centered variables of healthcare utilization, quality, and costs at periodic time intervals (10) for seven high-prevalence and high-cost diseases, including diabetes mellitus (11), COPD, myocardial infarction, congestive heart failure, stroke, major depressive disorder, and end-stage renal failure.

The aim of this study was to evaluate the utilization, quality, and costs of healthcare services used by patients with COPD in Iran, using data from IQCAMP. Our study provides direction on how to assess COPD care on a national level, particularly in countries that share similar healthcare systems to Iran. We also believe that this study can offer insight into the typical scenarios experienced by COPD patients within the Iranian healthcare architecture.

## Materials and methods

### Overview

This study is a part of the IQCAMP study, a national study aimed to affordably evaluate the quality and costs of healthcare across the country for seven high-prevalence and high-cost diseases in Iran from 2016 to 2018, including diabetes mellitus (11), COPD, myocardial infarction, congestive heart failure, stroke, major depressive disorder, and end-stage renal failure. This study reports first-hand data on patient experience regarding healthcare utilization, costs, and quality of care for each disease among a limited but representative patient sample. In the current study, we reported the utilization, quality and costs of healthcare for COPD across the country.

### Study design

The patients in the IQCAMP study were selected using a novel sampling method. A machine-learning-based sampling method was used to divide 31 provinces into eight clusters considering their similarity in healthcare structure and outcome data. One province from each cluster was selected for data collection. Simulation analysis of the sampling revealed an efficiency of up to 70% (10). One province per district was systematically selected for data gathering, and the decision tree machine learning method was applied for an accurate description of the features of selected clusters (Supplementary Figure S1 and Supplementary Table S1). More details are provided in depth elsewhere (10). Using the method, the sample size is calculated approximately 300 participants. In the selected clusters, eligible patients were identified by pulmonologists and were approached by trained study nurses to attract study participation.

COPD is medically defined and confirmed by spirometry via the forced expiratory volume (FEV1) to forced vital volume (FVC) ratio of below 0.7 (12). However, considering the paucity of spirometers and diagnostic equipment in scores of provinces in Iran, the study’s medical experts decided to use to use the physician confirmation of COPD cases instead of using the FEV1 measures. Notably, in case of availability, we also recorded spirometry results (12). Due to low service utilization of low severity cases, the IQCAMP team decided to fundamentally survey on patients with moderate-to-severe COPD, who were hospitalized at least once due to COPD exacerbation during the past 3 months. Disease severity was defined based on the pulmonologists’ expert opinion.

A group of nurses in each clusters called the participants and gave them detailed instruction about the study objectives and their right to leave the study at any time. The initial interview included participants’ past and current medical history. Then, three monthly follow-up interviews were held by the nurses to collect data on service utilization, quality indicators, and the cost of healthcare services received. The ethics committee of the Tehran University of Medical Sciences and the National Institute of Medical Research Development (NIMAD) approved the patient recruitment protocol.

### Variable and data collection

In this study, we aimed to gather three types of data: (1) the frequency of the utilization of healthcare services including inpatient and outpatient medical visits, medications, paraclinical services, and imaging, (2) quality of healthcare using quality indicators, and (3) direct and indirect costs of services provided by the patient, health insurance and government. A panel of medical experts for COPD, health economists, and epidemiologists designed the questionnaire based on their experience and literature review.

### Questionnaire

The questionnaire was face-validated and then updated after a pilot study with the participation of ten individuals. Finally, the study questionnaire was hosted on an android provisioned device and then underwent usability testing for the study nurses. The questionnaire included five main domains: (1) sociodemographics, (2) medical status, (3) healthcare utilization, (4) healthcare quality, and (5) healthcare costs.

The sociodemographic questionnaire consisted of questions about the gender, age, literacy, occupational status, and wealth index. The wealth index was used to divide the population into quintiles, whereby the first and fifth quintiles present the least fortunate and wealthiest households, respectively. Principal component analysis (PCA) was applied to derive the household wealth index based on questions on key dwelling characteristics and household ownership, as described in the study protocol (13). The health status questionnaire included questions regarding the current health condition of participants, their awareness about the medical condition, complications, and aftermaths of their disease, and smoking status.

Three major categories of services, including therapeutic, diagnostic, and patient support services, were included in the healthcare utilization questionnaire. Therapeutic services consisted of inpatient and outpatient visits; diagnostic services consisted of laboratory, testing, and imaging services; and patient support services included rehabilitation, home care, medical equipment utilization, and pharmacy services. In addition, all medications that were available in the Iranian Pharmacopoeia and were used to treat COPD were included in the survey.

To assess the quality of healthcare services, an expert panel of individuals involved in managing patients with COPD including primary care physician, internist, pulmonologist, and sub-specialist in COPD were gathered. The panel initially extracted the quality indicators for healthcare services from resources provided by the Ministry of Health and Medical Services of Iran, frameworks utilized in developing countries, and National Qualification Framework (NQF) developed for the United States (US) (14). Then, the panel conducted an extensive literature review and included other indices that were deemed essential based on their own experience in assessing the quality of care, but not considered in the guidelines mentioned earlier. Finally, a set of indicators with the highest rates of agreement upon were included as quality-of-care assessment indicators in this study. The quality indicators included 14 questions: seven questions for chronic phase of COPD, and seven questions for exacerbation episodes (acute phase). Smoking cessation advice, referral to smoking and tobacco quit centers, assessing the accuracy of inhaler utilization at the first visit and during the past year, vaccination against seasonal influenza and pneumococcal pneumonia, and considering spirometry during the past 12 months were indicators of chronic phase. Quality of care for exacerbation episodes was assessed by patient education about first measures during an exacerbation, oxygen therapy at the emergency department, assessment of the accuracy of inhaler usage after exacerbation episode, usage of glucocorticoids after discharge, referral to and completion of 4 week rehabilitation services after discharge, and pulse-oximetry during hospitalization. A list of all indicators assessed by medical experts to be included in the study is presented in Supplementary Table S2.

Two categories of healthcare costs included direct medical and indirect costs. The direct costs section included questions regarding healthcare diagnostic and therapeutic services costs, hospitalization costs, and costs attributable to medication, rehabilitation, and home services. The indirect costs section included direct non-medical costs (i.e., transportation, accommodation, and childcare costs during the hospitalization), absenteeism, loss of productivity, and wasted time during the doctor-patient visits. Considering the disparate sources of healthcare costs including out-of-pocket costs, health insurance share and the governmental support, direct medical costs were gathered via hospital invoices and self-declarative questionnaire.

Data were collected through patients’ medical records, health insurance details, and interviews of patients via structured questionnaires. Provisioned devices were used for the electronic migration of the structured questionnaire.

### Data analysis

The unit of analysis was defined as patient-month. Therefore, a 3 month follow-up of one patient was equal to three patient-month visits. Healthcare utilization data were reported by the number of events per month that resulted in healthcare services and the average number of monthly utilization of services per patient visit. To present the average annual costs of COPD, the average cost of each month was calculated and multiplied by 12. Purchasing Power Parity (PPP) was applied to convert Iranian Rials to US Dollars (USDs), where 1,000 USD equaled 16,773,000 Iranian Rials (15). Considering the importance of converting indirect costs to interpretable measures, firstly, the number of absent days from work due to their underlying disease multiplied in minimum daily wage to calculate absenteeism. Secondly, loss of productivity was calculated by asking the patients how much less they could earn due to their current illness. Quality of healthcare for COPD was assessed by reporting the number (%) of participants utilizing quality-of-care indices. All quantitative data were reported by central tendency (mean and median), and dispersion indices including standard deviation (SD) and interquartile range (IQR).

## Results

From 300 participants selected from eight clusters of Iran, a total of 235 (78.3%) patients with COPD completed the study, of which 154 (65.5%) were male. All patients have monthly been followed up for 3 months, which was interpreted as 705 patient visits. Sociodemographic characteristics of participants are presented in Table 1.

**Table 1:** Sociodemographic characteristics of participants.

Variable	*N* (%)
Sex	
Female	81 (34.5)
Male	154 (65.5)
Age group	
18–35 years	1 (0.4)
36–65 years	100 (42.6)
>65 years	118 (50.2)
Not defined	16 (6.8)
Education	
Hawza	1 (0.4)
Illiterate	57 (24.3)
Primary school	47 (20.0)
Middle or high school	21 (8.9)
High school graduate (Diploma)	26 (11.1)
University graduate	5 (2.1)
Post-graduate degree	2 (0.9)
Not defined	76 (32.3)
Household wealth index	
Very low	56 (23.8)
Low	71 (30.2)
Middle	61 (26.0)
High	31 (13.2)
Very high	16 (6.8)

Of all participants, 217 (92.3%) were aware of their disease, and 182 (83.9%) participants have been diagnosed with COPD for more than a year. Some 118 (50.0%) of participants declared they have a history of smoking, with 21 (17.8%) among women and 97 (82.2%) among men. Of participants with a history of smoking, 61 (51.7%) had not yet quit smoking [6 (10.0%) among women and 54 (88.5%) among men]. The mean (SD) and median (IQR) daily number of cigarette stubs were 21.1 (14.0) and 15.0 (20.0), respectively. The mean (SD) daily number of cigarette stubs among men was significantly higher than women: 21.0 (13.5) vs. 14.8 (16.1); *p*-value <0.001. The mean (SD) years of smoking was 31.9 (15.2) years, with 31.9 (15.1) and 22.8 (14.9) among women.

### Healthcare utilization

Table 2 presents the monthly utilization of various COPD services among 705 patient-visits. The most utilized services were pharmacy services with monthly use of 258 times (189 times for males and 69 times for females), followed by outpatient services with 142 times per month (99 times for males and 43 times for females). The most prescribed medications among patients with COPD were salbutamol inhaler [173 (73.6%)], tiotropium inhaler [98 (41.7%)] and ipratropium inhaler [95 (40.4%)]. The outpatient services were utilized less than four times a year. Almost none of the participants utilized the rehabilitation services.

**Table 2:** Monthly utilization of various services among 705 patient visits of COPD.

Services		Patients (No)	Event (No)	Event per patient
Therapeutic services				
Inpatient services	Total	42	48	0.11
	Female	18	20	0.13
	Male	24	28	0.10
Outpatient services	Total	112	142	0.32
	Female	29	43	0.27
	Male	83	99	0.35
Diagnostic services				
Laboratory services	Total	10	12	0.02
	Female	5	7	0.04
	Male	5	5	0.03
Imaging services	Total	26	29	0.07
	Female	4	4	0.03
	Male	22	25	0.09
Patient support services				
Rehabilitation services	Total	3	4	0.01
	Female	1	1	0.01
	Male	2	3	0.01
Pharmaceutics	Total	209	258	0.58
	Female	57	69	0.43
	Male	152	189	0.67
Homecare services	Total	2	2	0.00
	Female	1	1	0.01
	Male	1	1	0.00
Medical equipment	Total	18	18	0.04
	Female	5	5	0.03
	Male	13	13	0.05

### Healthcare quality

During the exacerbation phase of COPD, the blood oxygen levels of more than 80% of participants with exacerbated COPD were measured by pulse oximetry devices and documented, which was between 80 to 93% among the majority of hospitalized participants. In addition, more than 80% of eligible participants harmonically received oxygen therapy during hospitalization in the emergency departments. However, less than half of participants were educated about essential first measures during COPD exacerbation, which was much lower among female participants. Less than 70% of participants were evaluated for the precision of inhaler usage after the COPD exacerbation episode.

**Figure 1:**
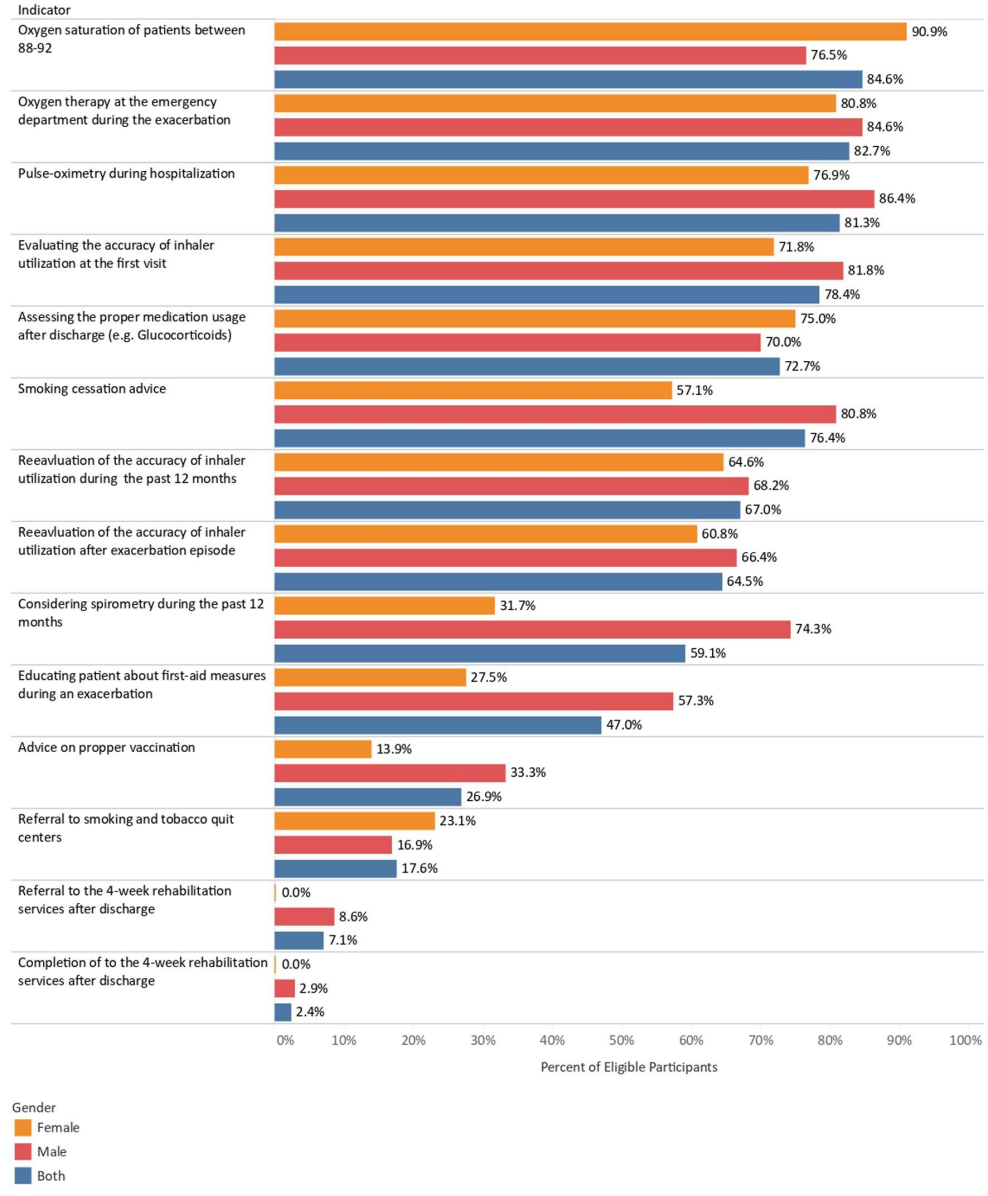
The quality indicators of COPD diagnosis and management among men and women.

Considering the chronic phase of COPD, some 60% of participants have been monitored by respiratory function tests and spirometry during the past year. Although the physician assessed the accuracy of inhaler usage among more than 75% of participants at the first patient-physician visit, only 67% of participants declared that the physician reassessed the accuracy during the past year. Most eligible participants were advised to quit smoking by their physicians. However, less than a third of participants were referred to smoking and tobacco quit centers and vaccinated against pneumococcus/Influenza. In addition, barely any participants were able to follow-up with rehabilitation services, and only 2% of participants have completed four-session rehabilitation services (Figure 1).

### Healthcare costs

The annual average direct cost of a patient with COPD was 1,605.5 USDs. Average out-of-pocket share of direct costs calculated 181.8 USD. While more than 85% of direct costs were due to hospitalization and inpatient services, most out-of-pocket costs were due to pharmacy services. Direct and indirect costs of COPD are presented in Table 3.

**Table 3:** Annual costs of various medical services for patients with COPD.

Services	**Out-of-pocket costs**			**Total costs**		
	Mean (SD)	Median (IQR)	Proportion (%)	Mean (SD)	Median (IQR)	Proportion (%)
Direct costs						
In-patient services	26.5 (712.3)	0.0 (1250.0)	14.6	1376.3 (45288.1)	7426.0 (43931.2)	85.7
Out-patient services	16.2 (159.6)	41.52 (256.7)	8.9	27.6 (169.7)	178.8 (280.4)	1.7
Laboratory services	2.8 (179.0)	214.7 (190.3)	1.5	6.2 (700.2)	400.7 (473.9)	0.4
Imaging services	7.4 (317.5)	185.3 (328.6)	4.1	10.4 (347.0)	236.6 (429.7)	0.7
Rehabilitation services	0.0 (N/A^a^)	N/A (N/A)	0.0	0.0 (0.0)	N/A (N/A)	0.0
Pharmaceutics	112.1 (523.8)	529.4 (514.6)	61.6	168.7 (1200.2)	572.4 (824.6)	10.5
Homecare services	4.8 (N/A)	2146.3 (0.0)	2.7	0.0 (N/A)	N/A (N/A)	0.0
Medical equipment	12.0 (1012.0)	1609.7 (1001.6)	6.6	16.2 (1352.4)	28611.8 (1287.8)	1.0
Indirect costs						
Non-health	N/A	N/A	N/A	854.8 (854.8)	400.7 (500.8)	18.0
Absenteeism	N/A	N/A	N/A	358.7 (358.7)	2.3 (535.2)	7.0
Loss of productivity	N/A	N/A	N/A	2679.5 (2679.5)	2146.3 (2146.3)	56.0
Time waste	N/A	N/A	N/A	933.0 (933.0)	310.9 (341.2)	19.0

a N/A: have not measured.

## Discussion

In this cohort study, we assessed the utilization, quality and costs of healthcare services for COPD in Iran using an affordable machine-learning based sampling method, which would pave the way for investigating such trends for high-volume and high-cost diseases on global scales and other nations. We found that pharmacy and outpatient medical services were the highest utilized healthcare services among patients with COPD. While medications imposed most of the out-of-pocket costs, the greatest portion of the direct costs were due to hospitalization and inpatient services. Healthcare quality provided for patients with COPD was higher in the exacerbation phases of the disease, and healthcare services revolved much less around chronic care for COPD.

Some 85% of the direct costs of COPD were due to hospitalization and inpatient services. Out-of-pocket costs were primarily due to pharmacy services. Enhanced availability of generic inhaled medication options could reduce cost and improve patients’ compliance (16). While COPD exacerbations generally result in healthcare resource use via increased physician visits, emergency department visits, hospitalizations, and pharmacy use (17), increased exacerbation frequency and severity impose higher costs, with the costs culminating among patients who require mechanical ventilation (18).

Healthcare providers measured patients’ blood oxygen levels in the emergency rooms and initiated oxygen therapy in most cases. Nevertheless, less than half of patients had been educated about the measures during COPD exacerbation including calling emergency services, administering oxygen supply, and maintaining in their most comfortable position. There is evidence that after the healthcare professionals have managed a COPD exacerbation episode of a patient, they have a golden opportunity to educate the patient about the necessary measures to prevent the following episode (19). Thus, patients need to receive appropriate maintenance therapy and attend appointments focusing on a medication regimen, proper inhaler technique, and assessment of symptoms to reduce exacerbation frequency and prevent the disease progression (18).

For further improvement in COPD course, it is recommended that pulmonary rehabilitation be started within 3 weeks of hospital discharge (20). Pulmonary rehabilitation is considered highly effective in improving dyspnea, exercise tolerance, and health-related quality of life. Some 60% of patients with COPD in England and Wales were enrolled into and completed a pulmonary rehabilitation program (21). Nevertheless, access to such services remains challenging for many patients, especially those living in limited resource settings (22). We found that only 7% of patients were advised by their physicians to attend rehabilitation programs within a month of referral. This might be partially due to scarcity of respiratory rehabilitating centers in Iran in addition to underestimation of the effectiveness of rehabilitation programs on COPD course. Unexpectedly, some 2% of patients reported completing on course of rehabilitation programs. In a study on Iranian patients with COPD, the barriers for pulmonary rehabilitation were partly due to patients lack of knowledge and the complexity and chronicity of COPD, which hinders patients from appropriate care. Another compelling reason regarding lower attendance of pulmonary rehabilitation services in Iran might be due to high costs of the service, inadequate insurance coverage, and inadequate professional competence of rehabilitation staff (23).

While spirometry remains the gold standard for diagnosis and monitoring COPD (24), less than 60% of the patients in this study were monitored by spirometry during the past year. In addition, we found that there was a significant two-fold gender gap between men and women in spirometry utilization. The underutilization of spirometry for COPD progression monitoring raises concerns, especially among women, who experience COPD exacerbations episodes more than men (25). COPD is mythically perceived as a male-dominated disease; however, it affects women and men almost equally (26). While smoking is traditionally considered the cause of COPD, household air pollution exposure and second-hand smoking are neglected risk factor for COPD, affecting women more than men (27). In addition, there is evidence that fear of stigmatization and improper knowledge about COPD cause could alter felt needs and healthcare-seeking behavior among women with COPD (28). On the other hand, primary care physicians play a central role in overseeing the multidisciplinary care of women with COPD (25). Thus, it is of paramount importance that physicians significantly improve awareness and encourage women with COPD to promptly seek medical advice. Primary care physicians play a central role in overseeing the multidisciplinary care of women with chronic obstructive pulmonary disease.

Smoking cessation is known to decrease mortality among patients with COPD via slowing the decline in lung function over time and limiting exacerbations (29). While physicians advised most patients to quit smoking, less than a third of participants were referred to professional centers for smoking cessation. Understandably, nearly half of the patients with a smoking history had not quit smoking at the study time. The accessibility to tobacco cessation centers is known to be a determinant of patients’ smoking cessation behavior (30). Iran’s Comprehensive National Tobacco Control Act has been in place since 2005. However, tobacco cessation programs in Iran have demonstrated no remarkable impacts. Notably, the prevalence of tobacco smoking has stagnated since 2004, when the STEPwise approach to chronic disease risk-factor surveillance (STEPS) survey was first conducted in the country (31). While the act also stated that smoking cessation counseling services ought to be provided in all health centers, the establishment of the national tobacco cessation helpline and tobacco cessation service clinics was announced in 2021 (32). Thus, the political will to lessen the burden of tobacco smoking needs to be strengthen, strict legislations need to be provided by the parliament and the government to discourage smoking, and more comprehensive solutions need to be employed to help quit smoking.

Less than a third of patients reported being vaccinated for pneumococcal and influenza during the last year, although vaccines are strongly recommended for patients with COPD for preventing poor outcomes (33). Reasons for low utilization of vaccines among patients could be improper knowledge (34), lack of vaccine recommendation by physicians, mistrust of vaccine safety, inconvenience of vaccination procedure, supply, and accessibility (35).

Patients with COPD often do not use their inhalers correctly, associated with an increased risk of disease exacerbation and hospitalization. Thus, periodic reinforcement of patient education on proper inhaler use is considered critical for COPD management (36). In our study, the accuracy of inhaler use was assessed by physicians for 75% of patients in the first visit and 70% after the last COPD exacerbation episode. This might partly be due to paucity of physician’s knowledge in addition to their negligence because of high workload in emergency rooms (37).

### Insights and future direction

Overall, based on the quality indicators assessed during the study, the focus of healthcare providers has been the management of the acute phases of COPD and missed the management of the chronic phase. This might be due to several reasons as follows.

Firstly, Iran has undergone significant changes in the burden of diseases pattern from communicable diseases to non-communicable diseases. Nevertheless, the healthcare system has not yet been aligned commensurate with such dramatic changes. After the Declaration of Alma-Ata on Primary Healthcare in 1978, rural and urban health centers were established with preventive practices. Considering that most of the Iranian population resided in rural areas at that time, most of the investment was allocated to rural areas (38). Notwithstanding, the rural primary healthcare system with trained community healthcare workers and well-established guidelines was effective in modifiable risk factors, including high systolic blood pressure and high plasma glucose (39). Nevertheless, the population distribution pattern in Iran changed dramatically in the preceding decades (40), while the network of urban health centers did not develop along with the growing demand due to civilization.

In the absence of a functional central healthcare system in urban areas, the primary healthcare services are decentralized and solo-practice. To establish an integrated healthcare system, the family physician program was launched in rural areas in 2005 with less than 20,000 population. The program introduced general practitioners to act as the focal point in the referral system. In 2012, the program was piloted in urban areas of two provinces (41). Although the family physician program could have had the capacity to provide the infrastructure required for the management of chronic non-communicable diseases, it failed due to improper design and mismanagement.

Thus, the current healthcare system lacks the integrated network of healthcare providers as needed for the long-term management of chronic non-communicable diseases.

Concurrently, the attitudes of insurance companies towards lessening the burden of diseases are still set for communicable diseases, the main characteristic of which is their acute onset. This has resulted in a narrow spectrum of the diversity of services and medications covered by insurance programs (38), which remains an obstacle to properly managing chronic diseases. Previous evidence suggests that limited insurance coverage for chronic disease management is ill-suited for the needs of patients with COPD and is associated with adverse clinical outcomes such as cost-related medication nonadherence, reports of foregone specialist and follow-up care, higher emergency room admission, and hospitalizations (42). Medication costs could be a contributing factor affecting patients’ adherence to prescribed medications. This is not confined to COPD and has also been witnessed in other diseases such as cancer (43) and cardiovascular disease (44). The potential inequities in quality of care across various income groups also need to be assessed in future studies.

Neglecting the long-term management of COPD could result in a higher frequency of COPD exacerbations. Frequent moderate-to-severe COPD exacerbation episodes are known to fuel the rate of lung function (45) and adversely affect the patients’ health-related quality of life. Holistic approaches towards COPD management could consist of multidisciplinary teams (18), close follow-ups, regular home visits (46), medication review, proper inhaler technique training, and education. Subsequently, COPD-related or all-cause hospital readmission rates (47) could be reduced, patients’ quality of care would improve, and direct or indirect costs would be reduced (18). Education should become an essential part of COPD management to empower patients to take control of their disease. Novel approaches could be employed to enhance the utilization of pulmonary rehabilitation among patients with COPD, such as using home-based nursing (48). Telehealth technology (49) could also be used to deliver pulmonary rehabilitation or even remote monitoring of proper inhaler usage (50). Such approaches could make rehabilitation more cost-effective and accessible, which need to be assessed in future studies.

### Strengths and limitations

IQCAMP provided a patient-centered disease-specific vision on healthcare utilization, quality, and costs for seven high-volume, high-cost diseases in Iran. Studies indicating national quality and costs of healthcare are mainly performed in countries with a well-established economy. In such settings, the real-world data to help understand patient experience usually come from claims, electronic medical records, electronic health records, and established data registries. Such surveys are usually limited to small samples collected by non-representative sampling methods in developing countries. In the meantime, large-sample studies could be extravagant for developing countries. IQCAMP is a cohort study that generates real-world data that provides first-hand information on patient experience for seven medical conditions that consume more than 50% of healthcare costs. Therefore, the sampling method in IQCAMP could empower policymakers to conduct small sample size surveys with celerity and at a low cost.

We also acknowledge the limitations of the study. Although the data collection method of the study is more advantageous than the convenience sampling method, it is not as representative as simple random sampling at a national level. This could call for further research to investigate the optimum sampling method in health systems research to address the healthcare utilization, quality, and costs for COPD, as studied in the present research. It is worth mentioning that planning, conducting, and implementing the current survey are too tedious and have many complexities, hindering the study’s repetition. Nevertheless, the study design was deemed rational in Iran’s lack of an integrated health data system. As data on healthcare utilization was partly based on self-report, it could be subject to recall bias. In addition, the medication count in patients’ prescriptions was used as a proxy of medication utilization, which could not reflect the patients’ adherence to treatment. We were also eager to investigate the sub-national inequities in quality of care and utilization; however, the study did not have enough power for subgroup analysis and determination of geographical distribution due to a limited number of cases.

## Conclusion

Although COPD is a high-cost and high-volume disease in Iran, healthcare quality provided for patients with COPD was solely high in the exacerbation phases of the disease, and healthcare services revolved much less around chronic care for COPD. Iran’s healthcare system is in transition from communicable diseases to non-communicable diseases. More concentration on post-acute phases of NCDs is required to contain NCDs pandemic.

## Data availability statement

The raw data supporting the conclusions of this article will be made available by the authors, without undue reservation, upon request.

## Ethics statement

The current study is a joint product of two grants, IranMHS and IQCAMP, that were approved by the Ethics Committee of Tehran University of Medical Sciences in Iran (no. IR.TUMS.REC.1394.1900) and the ethics committee of Iran National Institute for Medical Research Development, Tehran, Iran (Ethics code: IR.NIMAD.REC.1395.003), respectively. The patients/participants provided their written informed consent to participate in this study.

## Author contributions

SK and SaS: conceptualization. FM, MM, NS, YF, ShS, SK, and SaS: data curation. MA-K, S-HG, NA, YF, MA, SR, ShS, and SaS: formal analysis. SaS: funding acquisition. MA-K, YF, ShS, NR, SK, and SaS: investigation. FM, YF, MA, ShS, NR, SK, and SaS: methodology. FM, YF, ShS, NR, and SaS: project administration. NR and SaS: resources. YF, MA, HZ, MK: software. YF, NR, and SaS: supervision. YF, NR, SK, and SaS: validation. S-HG, MA-K, and SaS: writing—original draft preparation. S-HG, FM, MA-K, SP, BR, NA, YF, ShS, NS, MM, MA, HZ, MK, RS, M-RM, SR, NR, MF, MS, ZA, SK, and SaS: writing—review and editing. All authors contributed to the article and approved the submitted version.

## Conflict of interest

The authors declare that the research was conducted in the absence of any commercial or financial relationships that could be construed as a potential conflict of interest.

## Publisher’s note

All claims expressed in this article are solely those of the authors and do not necessarily represent those of their affiliated organizations, or those of the publisher, the editors and the reviewers. Any product that may be evaluated in this article, or claim that may be made by its manufacturer, is not guaranteed or endorsed by the publisher.
