# Supplementary material for: Patient experience with chronic obstructive pulmonary disease: a nationally representative demonstration study on quality and cost of healthcare services
Source: Front Public Health. 2023 Jun 15;11:1112072. doi: 10.3389/fpubh.2023.1112072 (PMC10308222; doi:10.3389/fpubh.2023.1112072)
Supplement: Supplementary file 1 [file Table_1.DOCX]

# Appendix 1

## Supplement to

First National Survey on Healthcare Utilization, Quality, and Costs for Chronic Obstructive Pulmonary Disease in Iran: Patient Journey

## Supplementary Figure 1

## Supplementary Table 1

Various districts of the country were divided into eight clusters based on the healthcare structure and outcome data of different districts using hierarchical and model-based clustering methods. One province per district has been systematically selected for data gathering, and the decision tree machine learning method was applied for an accurate description of the features of selected clusters. Clusters and selected provinces were provided in Supplementary Figure 1 and Supplementary Table 1.
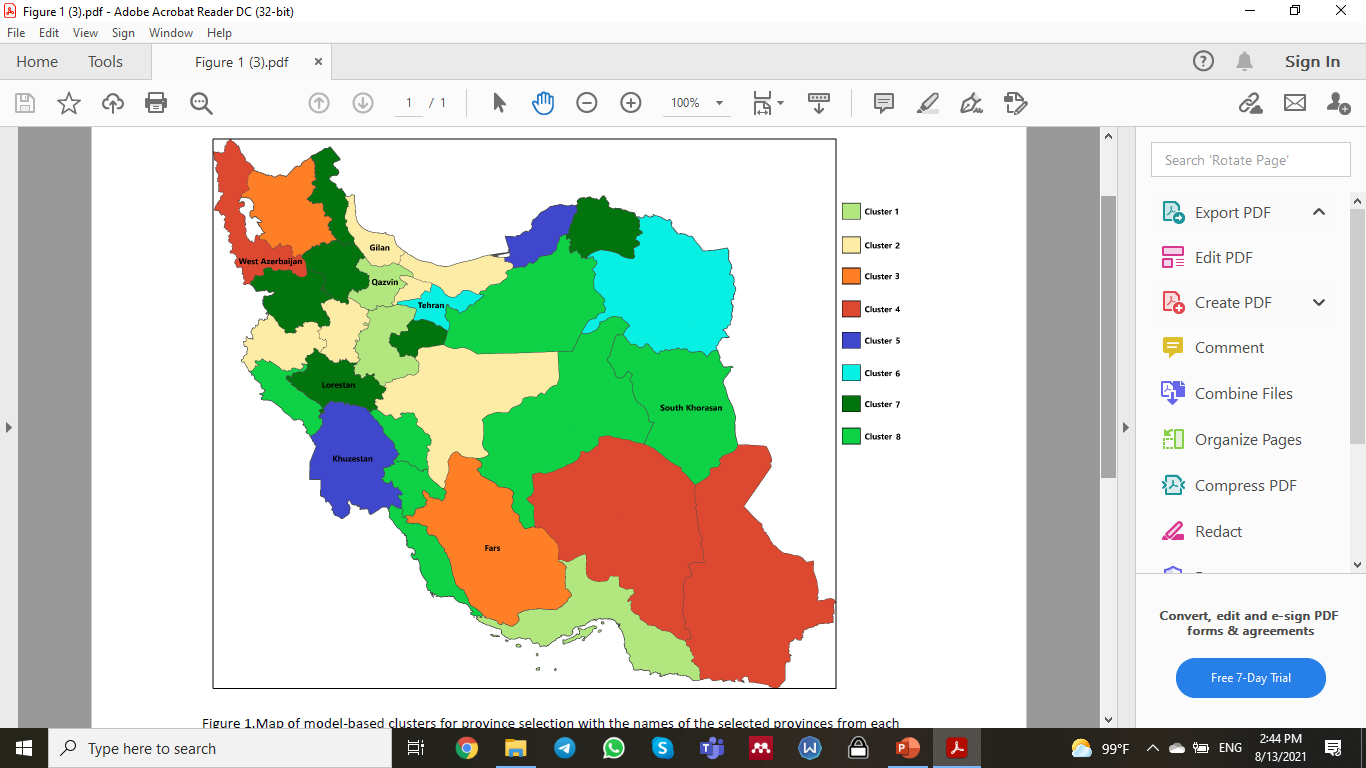


| **Supplementary Table 1:** Clusters and selected province for IQCAMP study in Iran | | |
| --- | --- | --- |
| **Cluster** | Provinces | Selected province |
| Cluster 1 | Qazvin, Markazi, Hormozgan | Qazvin |
| Cluster 2 | Gilan, Mazandaran, Kermanshah, Isfahan, Hamedan, Alborz | Gilan |
| Cluster 3 | East Azerbaijan, Fars | Fars |
| Cluster 4 | West Azerbaijan, Kerman, Sistan and Baluchestan | West Azerbaijan |
| Cluster 5 | Khuzestan, Golestan | Khuzestan |
| Cluster 6 | Tehran, Razavi Khorasan | Tehran |
| Cluster 7 | Kurdistan, Lorestan, Zanjan, Ardabil, Qom, North Khorasan | Lorestan |
| Cluster 8 | Chaharmahal and Bakhtiari, Ilam, Kohgiluyeh and Boyer-Ahmad, Bushehr, Semnan, Yazd, South Khorasan | South Khorasan |

## Supplementary Table 2

Quality indicators were gathered through literature review, which medical experts assessed to form the IQCAMP quality indicators.

| **Supplementary Table 2**: List of quality indicators assessed with the medical experts for IQCAMP survey of chronic obstructive pulmonary disease (COPD) |
| --- |
| Chronic phase |
| 1. The percentage of patients diagnosed by spirometry, including reversibility testing for newly diagnosed patients. |
| 2. The percentage of all patients with COPD in whom diagnosis has been confirmed by spirometry, including reversibility testing. |
| 3. The percentage of patients with COPD in whom there is a record of smoking status. |
| 4. The percentage of patients with COPD who smoke, whose notes contain a record of smoking cessation advice. |
| 5. The percentage of patients with COPD who smoke, whose notes contain a record of a referral to smoking cessation specialist service. |
| 6. The percentage of patients with COPD in whom there is a record of tobacco smoke. |
| 7. The percentage of patients with COPD who smoke tobacco, whose notes contain a record of a referral to tobacco smoking cessation specialist service. |
| 8. The percentage of patients with COPD who smoke and receiving pharmacological interventions for smoke cessation. |
| 9. The percentage of patients with COPD with a record of FEV1. |
| 10. The percentage of patients with COPD receiving short-acting inhaled treatment. |
| 11. The percentage of patients with COPD receiving short-acting inhaled treatment in whom there is a record that inhaler technique has been checked. |
| 12. The percentage of patients with COPD receiving long-acting inhaled treatment. |
| 13. The percentage of patients with COPD receiving long-acting inhaled treatment in whom there is a record that inhaler technique has been checked. |
| 14. The percentage of patients with COPD receiving inhaled combination therapy. |
| 15. The percentage of patients with COPD receiving inhaled combination therapy in whom there is a record that inhaler technique has been checked. |
| 16. The percentage of patients with COPD who have had influenza immunization. |
| 17. The percentage of patients with COPD who have had pneumococcal immunization. |
| 18. The percentage of patients with COPD who were undergone oral corticosteroid reversibility tests |
| 19. The percentage of patients with COPD using a hand-held inhaler device (including a spacer device). |
| 19. The percentage of patients with COPD in whom there is a record that inhaler technique has been re-checked regularly. |
| 20. The percentage of patients with COPD treated with long-term oral corticosteroid therapy should be monitored for the development of osteoporosis. |
| 21. The percentage of patients with COPD treated with long-term oral corticosteroid therapy in whom there is a record of monitoring for the development of osteoporosis. |
| 22. The percentage of patients with COPD who were regularly evaluated for receiving long-term oxygen therapy with spirometry, arterial blood gas, and physical examination. |
| 23. The percentage of patients with COPD in whom there was a record of pulmonary edema assessment. |
| 24. The percentage of patients with COPD in whom there was a record of pulmonary rehabilitation programs. |
| 25. The percentage of patients with COPD in whom there was a record of completing pulmonary rehabilitation programs. |
| 26. The percentage of patients with COPD who were evaluated by spirometry at least once in the previous year. |
| 27. The percentage of patients who were evaluated for malnutrition using body mass index. |
| Exacerbation phase |
| 1. The percentage of patients with COPD who were hospitalized with the diagnosis of COPD exacerbation. |
| 2. The percentage of patients with COPD exacerbation history in whom there was a history of pulse oximetry in the emergency department at admission time. |
| 3. The percentage of patients with COPD exacerbation history in whom the sputum samples were sent for culture. |
| 4. The percentage of patients with COPD exacerbation history who were undergone chest radiography. |
| 5. The percentage of patients with COPD exacerbation history who were assessed with ECG. |
| 6. The percentage of patients with COPD exacerbation history managed by a multi-professional team including nurses, physiotherapists, occupational therapists, and generic health workers. |
| 7. The percentage of patients with COPD exacerbation history who were evaluated for their inhaler usage. |
| 8. The percentage of patients with COPD exacerbation history who are considered for rehabilitation services at discharge. |
| 9. The percentage of patients with COPD exacerbation history who were educated for first-aid measures during the exacerbation time. |
| 10. The percentage of patients with COPD exacerbation history receiving oxygen therapy in the emergency department. |
| 11. The percentage of patients with COPD exacerbation history assessed the medication usage during discharge. |
